# The Healthiness of Packaged Food and Beverage Products in the Kingdom of Saudi Arabia

**DOI:** 10.3390/nu17111895

**Published:** 2025-05-31

**Authors:** Elizabeth K. Dunford, Reem F. Alsukait, Majid M. Alkhalaf, Mariam M. Hamza, Mohammed A. Shahin, Volkan Cetinkaya, Taghreed Alghaith

**Affiliations:** 1 Department of Nutrition, Gillings Global School of Public Health, The University of North Carolina at Chapel Hill, Chapel Hill, NC 27599, USA; 2 Food Policy Division, The George Institute for Global Health, University of New South Wales, Sydney, NSW 2000, Australia; 3 Community Health Sciences, College of Applied Medical Sciences, King Saud University, Riyadh 11451, Saudi Arabia; rasukait@ksu.edu.sa; 4 Public Health Authority, Riyadh 13351, Saudi Arabia; mmkhalaf@pha.gov.sa (M.M.A.); mashahin@pha.gov.sa (M.A.S.); tmghaith@pha.gov.sa (T.A.); 5 World Bank Group, Washington, DC 20433, USA; mhamza@worldbank.org (M.M.H.); vcetinkaya@worldbank.org (V.C.)

**Keywords:** packaged foods, nutrient profiling, Nutri-Score, high fat sugar salt, nutrient profile model

## Abstract

Background/Objectives: In 2020, the National Nutrition Committee in Saudi Arabia launched a nutrient profile model, aiming to support the classification of foods and beverages in line with successful international approaches. The objective of this study was to compare the existing Saudi Arabian nutrient profile model to other established models to help inform diet-related policies in the country. Methods: Packaged food and beverage data were obtained from Mintel’s Global New Products Database. Products were evaluated under the Saudi Arabian nutrient profile model, Nutri-Score and Chile’s high fat, salt, sugar (HFSS) model. Agreement among the three nutrient profile models was examined using Fleiss’ kappa statistic. Results: There were 6940 products used in analysis. All three models showed a low proportion of eligible/healthy products, with 26% for Chile’s HFSS model, 28% for Nutri-Score and 25% for the Saudi Arabian nutrient profile model. There was substantial agreement (86%; k = 0.74) among all three models examined, with the highest agreement between the Saudi Arabian nutrient profile model and the Nutri-Score model. Conclusions: All three demonstrated a sub-optimal level of overall healthiness in the Saudi Arabian packaged food and beverage supply, with <30% of products under all models considered “healthy”. Given the substantial agreement among all three nutrient profiling approaches examined, it is likely that Saudi Arabia could benefit from the use of a categorical approach to nutrient profiling such as the Nutri-Score model, which allows for a more scaled view on product healthiness compared to a binary approach.

## 1. Introduction

The Kingdom of Saudi Arabia is one of the world’s fastest-growing economies. Recent increases in household income have been accompanied by a nutritional transition, with westernized diets replacing traditional diets, and the population overall is becoming less active [1,2]. This shift towards a more westernized dietary pattern consisting of more processed foods and lower levels of physical activity has been associated with higher rates of non-communicable diseases [3]. In fact, data from the Global Burden Of Disease study have indicated that Saudi Arabia has some of the highest levels of obesity and diabetes in the world (42% for women and 31% for men) [4]. Diet-related chronic diseases such as type 2 diabetes, cardiovascular diseases and obesity are major contributors to the global burden of disease [5]. The prevalence of these diet-related diseases is known to rise in conjunction with higher consumption of processed foods [6]. Compared to unpackaged products, processed packaged foods and beverages can be higher in sodium, added sugars, excess energy and saturated fat [7]. Therefore, improvements in the healthiness of packaged foods and beverages could support a reduction in diet-related disease through decreased consumption of such nutrients of concern [8].

Nutrient profiling is a method of or ranking or classifying foods and beverages according to their nutritional composition. It also provides a way to evaluate the healthiness of food and beverage products. Nutrient profiling is designed to evaluate individual foods (not diets), yet nutrient profile models are often used to underpin national policies aiming to improve the nutritional quality of the overall food supply [9]. Nutrient profiling is recognized by the World Health Organization (WHO) as a useful approach to use in combination with other interventions that improve diet quality [10]. There is no consensus on whether one nutrient profiling model is better than another. The WHO catalogue on nutrient profile models lists >65 existing nutrient profiling models; however, this document is now outdated [9]. An updated literature review found that 78 models have been developed since the WHO catalogue was released [10]. These include government-led models such as Chile’s warning labels [11], government endorsed models like France’s Nutri-Score [12] and industry-led schemes such as the Choices International Programme [13], as well as models developed for use internally by food and beverage companies.

In 2020, the National Nutrition Committee in Saudi Arabia launched a nutrient profile model, aiming to support the classification of foods and beverages in line with successful international approaches. The resulting model was based heavily on the Ofcom model from the UK and results in a binary outcome of “healthy” or “not healthy” [14]. However, this model has yet to be applied to the Saudia Arabian packaged food supply, and it has not been examined against other existing validated nutrient profile models. As such, the objective of this study was to compare the existing Saudi Arabian nutrient profile model to other established models to help inform diet-related policies in the country.

## 2. Materials and Methods

Packaged food and beverage data for this project derived from Mintel’s Global New Products Database [15]. All packaged food and beverage products introduced to the Kingdom of Saudi Arabia market between 2014 and 2024 were included in analysis. Duplicate products (i.e., products with identical barcodes or product names) were excluded. Sales data from Euromonitor Passport [16] were used to weight overall results by category-level sales.

For this project, packaged food and beverage products were compared to the local Saudi Arabian nutrient profile model as well as the Nutri-Score nutrient profile model and Chile’s high fat, salt, sugar (HFSS) approach for identifying less healthy foods.

Saudi Arabian nutrient profile model: The government of Saudi Arabia developed their nutrient profile model as a tool to assist in identifying less-healthy foods and beverages [17]. The model is based heavily on the original Ofcom nutrient profiling model from the UK [14], with points given for “negative” nutrients such as saturated fat, calories, sodium and total sugar, which are then offset with “positive” components such as the proportion of protein, fiber and fruits/vegetables/nuts. Products with a score of <4 for foods and <1 for beverages are classified as healthier options.

Chile’s high fat, salt, sugar (HFSS) nutrient profiling approach: The Chilean government was the first country in the world to have a nutrient profile model enacted into law (in 2016). The law applied to front-of-pack warning signs, taxation and marketing restrictions. The nutrient thresholds were set to become stricter over three separate implementation dates [11]. The Phase 2 criteria were used for this report because they have been used by other countries as final thresholds (Table S1). Beverages and foods were classified as not meeting the criteria if they contained added sugar, added sodium or added saturated fat ingredients and exceeded the nutrient thresholds.

Nutri-Score: Nutri-Score is a nutrient profile model that provides a rating on the healthiness of food and beverages, using colors to place food products into one of five categories: from category A (dark green; higher nutritional quality) to category E (dark orange; lower nutritional quality) [12]. Nutri-Score was developed to support consumers in identifying healthier food and beverage products and therefore support a reduction in nutrition-related chronic diseases. The score for a product is calculated by allocating points for nutritional content per 100 g/mL of energy, total sugar, saturated fat, sodium, protein, dietary fiber and fruits/vegetables/legumes. An update to the original Nutri-Score algorithm was released in 2023 [12], with this used for analysis in the current study.

Data from Mintel’s Global New Products Database were categorized into one of 27 Euromonitor Passport categories to examine sales trends and to weight overall results by category-level sales. Results were calculated overall and by Euromonitor Passport category. Products were assigned to either ‘foods’ or ‘beverages’ under the Saudi Arabia nutrient profile model and Chile’s HFSS nutrient profile model and placed in one of five categories (general foods, beverages, fats, red meat, cheese) for the Nutri-Score analysis.

Products were flagged as outliers if they were above or below 5 standard deviations from the overall mean for total sugars, sodium, saturated fat or energy content. However, visual examination of each outlier flagged was undertaken to determine whether it should be removed from analysis. Category-level distributions were also examined and each potential outlier screened prior to removal. The proportion of food and beverage products meeting criteria for each nutrient profile model was examined. Results were examined overall and by Euromonitor Passport category. Results were also weighted using category-level 2023 sales data from Euromonitor Passport in secondary analyses where applicable. Agreement among the three nutrient profile models was examined using Fleiss’ kappa statistic. As Nutri-Score is not a binary outcome, one was created by defining “unhealthy” as products with the least healthy ‘D’ and ‘E’ ratings, in line with previous research [18]. All analyses were undertaken using Stata statistical software, V18.

## 3. Results

Initially, 14,047 products were extracted from Mintel’s Global New Products Database in April 2024. Products were excluded from all analyses if they were from prior to the year 2014 (*n* = 6515), if they were duplicate barcodes (*n* = 161) and if they were duplicate product names (*n* = 154), leaving *n* = 6940 products for analysis. Information on missing data is provided in Table S2, showing 30% of products did not label total sugar values, 28% did not label saturated fat values, and 26% did not label sodium values.

### 3.1. Saudi Arabian Nutrient Profile Model

**Figure 1:**
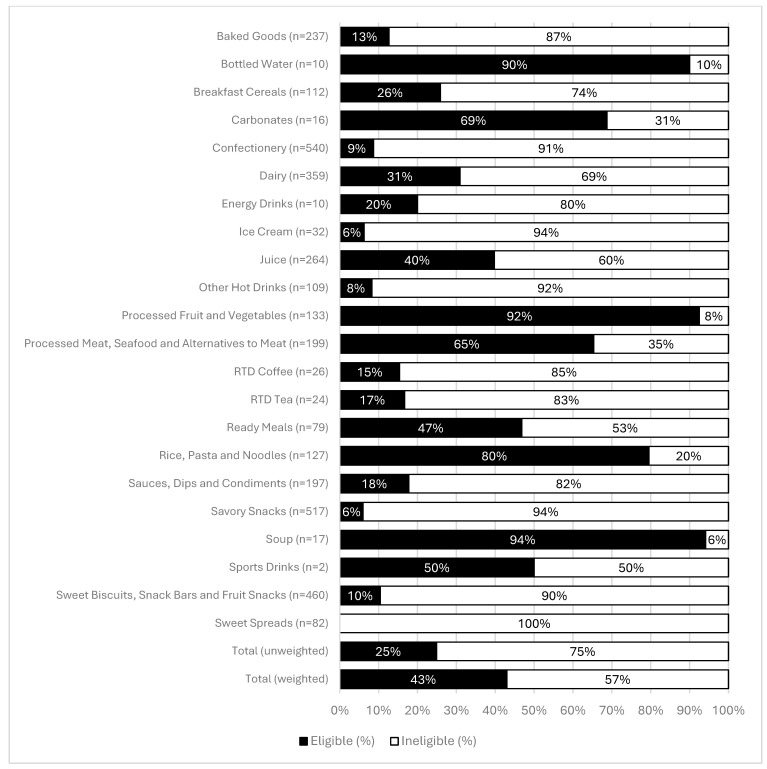
Proportion of Saudi Arabian packaged food and beverage products eligible under the Saudi Arabian nutrient profile model, by category. “Eligible” is defined as food products with a score of <4 and beverage products with a score of <1.

Out of *n* = 6940 products, *n* = 2902 (42%) were missing complete nutrient values required for analysis under the Saudi Arabian nutrient profile model (energy, saturated fat, total sugar, sodium and/or protein). An additional *n* = 486 were products that were not covered by the Saudi Arabian model (e.g., baby foods). As a result, there were *n* = 3552 products examined under the Saudi Arabian nutrient profile model. The number of products ranged from *n* = 2 for Sports Drinks to *n* = 540 for Confectionery (Figure 1). Overall, 25% of all products examined met the eligibility criteria under the Saudi Arabian nutrient profile model (Figure 1). This proportion increased once the data were weighted by category sales, resulting in 43% being eligible. This indicates that healthier products represented higher proportions of sales, increasing the overall proportion eligible (i.e., considered ‘healthy’) under the Saudi Arabian model. Beverage products had a higher proportion of products (46%) meeting the nutrient criteria under the Saudi Arabian nutrient profile model compared to foods (31%; Figure S1). Soup was the category with the largest proportion of eligible products (94%) followed by Processed Fruit and Vegetables (92%) and Bottled Water (90%). The categories with the smallest proportion of eligible products were Ice Cream (6%), Savory Snacks (6%) and Sweet Spreads (0%). Only six out of the twenty-two Euromonitor categories examined had more than half of all products considered healthy under the Saudi Arabian nutrient profile model.

### 3.2. Nutri-Score

**Figure 2:**
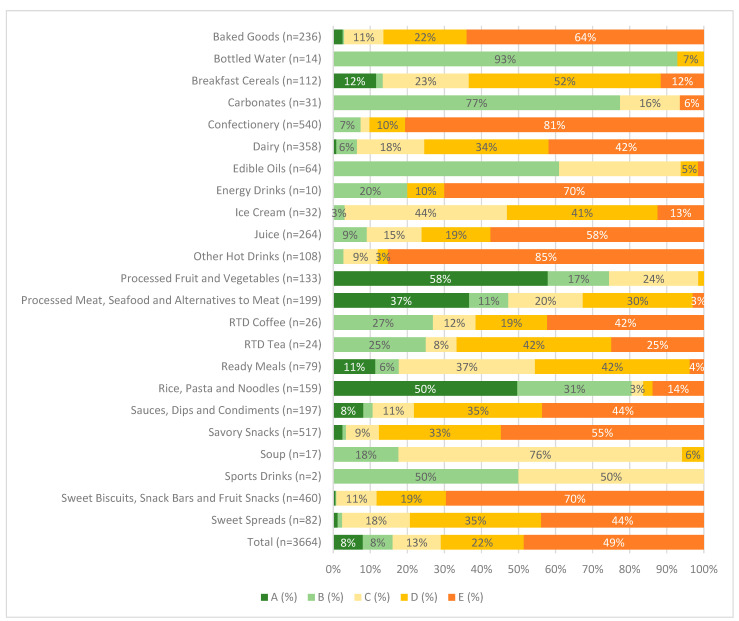
Proportion of Saudi Arabian packaged food and beverage products receiving each rating under the Nutri-Score nutrient profile model, by category. Each color represents a level of healthiness from category A (dark green; higher nutritional quality) to category E (dark orange; lower nutritional quality).

Out of *n* = 6940 products, *n* = 2902 (33%) were missing nutrient values required for analysis under the Nutri-Score nutrient profile model, and *n* = 374 products were specified to be excluded under the Nutri-Score model. As a result, there were *n* = 3664 products examined. The number of products ranged from *n* = 2 for Sports Drinks to *n* = 540 for Confectionery (Figure 2). Beverage products had a higher proportion of products receiving an ‘E’ (least healthy) rating under Nutri-Score (56%) compared to foods (47%; Figure S2), and beverages also had 0% of products receiving an ‘A’ (most healthy) rating. Just under half of all products in total, (49%) had a Nutri-Score of ‘E’ (least healthy), ranging by category from 0% of Processed Fruit and Vegetables, Sports Drinks and Bottled Water to 85% of Other Hot Drinks (Figure 2). Only 8% of products received a Nutri-Score of ‘A’ (most healthy). The categories with the largest proportion of products receiving a Nutri-Score of ‘A’ were Processed Fruit and Vegetables (58%), Rice, Pasta and Noodles (50%) and Processed Meat, Seafood and Alternatives to Meat (37%). Eleven of the 22 categories had zero products receiving a Nutri-Score of ‘A’.

### 3.3. Chile’s HFSS Nutrient Profiling Model (Phase 2)

**Figure 3:**
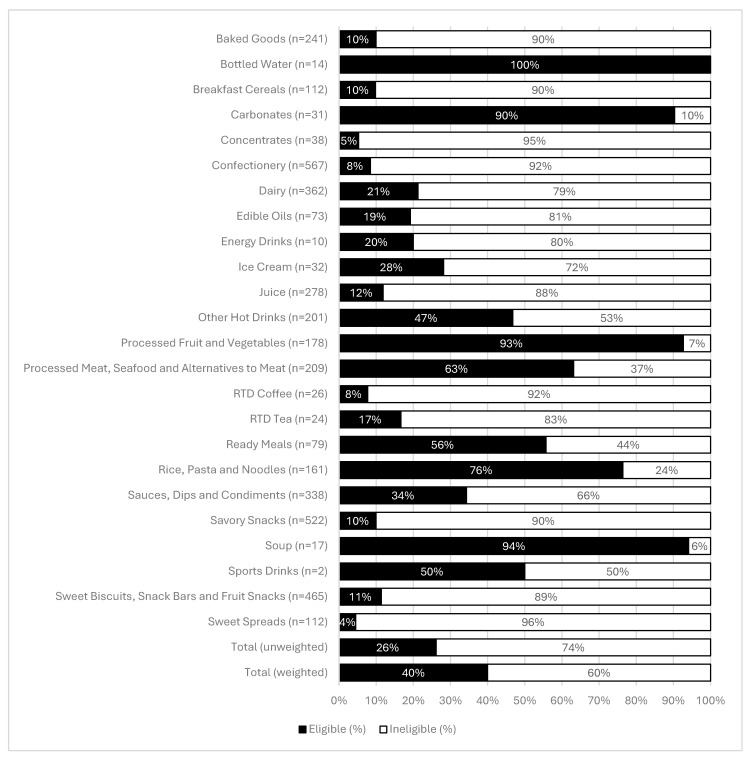
Proportion of Saudi Arabian packaged food and beverage products meeting nutrient and ingredient criteria under the Chilean HFSS model, by category. “Eligible” is defined as food and beverage products that did not exceed nutrient thresholds and did not contain added sugar, added sodium or added saturated fat ingredients.

Out of *n* = 6940 products, *n* = 2779 (40%) were missing nutrient values required for analysis under Chile’s HFSS nutrient profiling model, and an additional *n* = 69 products (baby foods) were not suitable to be evaluated using this model. As a result, there were *n* = 4092 products examined. The number of products ranged from *n* = 2 for Sports Drinks to *n* = 567 for Confectionery (Figure 3). Overall, 26% of all products were considered eligible (i.e., not HFSS) under this model, increasing to 40% once results were weighted by category sales. Foods and beverages both had similar proportions eligible under this model (Figure S3), with food having 27% and beverages 30%. Bottled Water had the lowest proportion of HFSS products (0%), followed by Soup (6%) and Processed Fruit and Vegetables (7%) (Figure 3). Sweet Spreads had the highest proportion of HFSS products (96%) followed by Concentrates (95%).

### 3.4. Agreement Among the Three Nutrient Profile Models

Out of *n* = 6940 products, there were *n* = 3548 products that had sufficient data to be examined under all three nutrient profile models. Table 1 shows the number of products by Euromonitor category included in this part of the analysis. There was substantial agreement among all three nutrient profile models (k = 0.74; Table 2), with 86% of all products being rated the same under all three models. The highest agreement was seen in Energy Drinks and Soup, meaning that all three nutrient profile models rated products in these categories in the same way (as Fleiss’ k = 1.00 for both categories). The lowest agreement was seen in Sports Drinks and Ice Cream, meaning that these products were rated differently among the three nutrient profile models. Tables S3–S5 show results comparing each model with another. Nutri-Score showed the highest agreement with the Saudi Arabian nutrient profile model, with k = 0.78 and 91% agreement. There was at least 75% agreement for all but two of the twenty-two categories included. Chile’s HFSS model compared to the Saudi Arabian model was not far behind, with k = 0.72 and 89% agreement and also at least 75% agreement for all but two of the included categories.

## 4. Discussion

This study represents the first comprehensive overview of the overall healthiness of the Saudi Arabian packaged food supply using established nutrient profile models. All three nutrient profile models examined in this study demonstrated a sub-optimal level of overall healthiness in the Saudi Arabian packaged food and beverage supply, with 26% for Chile’s HFSS model, 28% for Nutri-Score and 25% for the Saudi Arabian nutrient profile model considered ‘healthy’. Results were mainly in line with research from other countries. For example, research examining the US packaged food supply found that 74% of products were considered ineligible under the Chilean HFSS approach [19], matching the results found in this report for Saudi Arabia. When it comes to Nutri-Score, results for Saudi Arabia appear to be a little worse than what has been reported in other countries [20], with 49% of all Saudi products receiving an ‘E (least healthy) rating. However, previous studies have also used the original Nutri-Score algorithm and not the updated algorithm, making comparisons less useful. It will be important to follow future research from other countries using the updated Nutri-Score algorithm to understand how the Saudi Arabian food supply can be compared.

Results demonstrated that there was substantial agreement among all three models examined, with 86% of products rated the same under each model. Both the Saudi Arabian nutrient profile model and the Chilean HFSS model are binary approaches to determine whether packaged food and beverage products are deemed “eligible” or “ineligible” (healthy versus unhealthy). Nutri-Score, on the other hand, assigns products to one of five ‘categories’ (A–E), allowing for results to be understood using more of a scaled approach. For example, under the Saudi Arabian nutrient profile model, 53% of Ready Meals were considered ineligible/unhealthy. However, under Nutri-Score, the spread of healthiness was made clearer, with only 4% of products receiving the ‘least healthy’ score of ‘E’, 42% receiving a score of ‘D’ and an additional 37% receiving a score of ‘C’. Binary systems can often result in a very low proportion of the food supply meeting eligibility (healthiness) criteria [18] compared to category-based approaches, and so depending on the policy outcome in question, different approaches may yield more useful outcomes. It may be that due to the high agreement among all three models examined, a category-based approach such as Nutri-Score may be most useful in supporting front-of-pack labelling initiatives in the Kingdom of Saudi Arabia compared to binary approaches that do not indicate how healthy or unhealthy a product is. This idea is further supported by recent research demonstrating that Nutri-Score improved the healthiness of shopping baskets in an online shopping trial in Saudi Arabia more than Chile’s warning label approach [21].

Given that research has demonstrated that total intake of added sugar is extremely high in the Kingdom of Saudi Arabia (73 g/day) [22], it is concerning that so many products (30%) were missing total sugar content on product labels. It is unknown whether these missing values were due to data entry errors or due to limitations in the labelling requirements and legislation in the Kingdom of Saudi Arabia at the time when data were captured. A similar concern is that sugar-sweetened beverages are the largest contributor to sugar intake in Saudi children [23], and Carbonates was one of the top-selling categories examined in this report (representing 10% of total packaged food and beverage sales). With total sugar not being consistently available on nutrition labels in the Kingdom of Saudi Arabia, consumers are unable to make an informed choice when it comes to the healthiness of foods and beverages they are purchasing. However, research has shown that only 29% of Saudis check the nutrition label prior to making a purchase (versus more than 50% checking the expiration date or production date) [24], and so it may also be important to consider front-of-pack labelling approaches for the Kingdom of Saudi Arabia to ensure consumers are better informed. It is also possible that recent changes to labelling legislation such as the inclusion of added sugar (released in 2021) will ensure that in the future, more products will have complete nutritional labelling on-pack.

Baked Goods was the category contributing the largest proportion of overall sales in 2023 in the Kingdom of Saudi Arabia (26%). Baked Goods also had a very high proportion of products considered unhealthy under each nutrient profile model examined (ranging from 64% to 90%), and so, from a policy perspective, it will be important to carefully monitor products in this category. Similarly, careful monitoring of the sale and consumption of sugar-sweetened beverages will be critical given both the high sugar intake in the Saudi population, as well as these products representing a large proportion (10%) of all packaged food and beverage sales.

Although this study is the first in-depth review of the healthiness of the overall packaged food and beverage supply in Saudi Arabia, there is one previous study that has examined the healthiness of a sample (*n* = 1153) of Saudi Arabian packaged food and beverage products [25]. This study found that 46.9% of products displaying health or nutrition claims were less healthy (under the UK Ofcom model, which is very similar to the Saudi Arabian model) than those not carrying claims. It will be an important part of future research in this area to ensure that the use of health and nutrition claims is monitored in tandem with the levels of nutrients of concern in packaged foods and beverages in the Kingdom of Saudi Arabia.

This study has limitations. Data from Mintel’s Global New Products Database were subject to errors, and although efforts were made to remove erroneous data from analyses, there is always the chance that some data were incorrect. However, these errors would be unlikely to change the interpretation of the overall results of this report. Mintel’s Global New Products Database provides information for food and beverages that are either new to supermarket shelves or that are products that have been reformulated. Hence, it is possible that some products that have remained on the Saudi Arabian market for beyond the ten years captured through this analysis were not captured. Euromonitor Passport category-level sales data are not provided for every individual product. As such, it is unknown what weighting each product within each category represents. Additionally, foods and beverages purchased and consumed outside the home, such as from fast food outlets or restaurants, were not included in this study but represent an important component for consideration in future research and food policies in Saudi Arabia.

## 5. Conclusions

With the plethora of nutrition-related interventions being launched in the Kingdom of Saudi Arabia [26] and potentially more to come, it will be imperative to monitor the healthiness of the packaged food and beverage supply. Given the substantial agreement among all three nutrient-profiling approaches examined, the Kingdom of Saudi Arabia would benefit from the use of any of the three nutrient profile models examined in this study. As a nutrient-profiling approach that allows for a scaled view on product healthiness, Nutri-Score could be a useful option for front-of-pack labelling, especially given that recent research has shown it to be more effective at improving consumer shopping choices than Chile’s warning label. Future research would benefit from examining consumer preference in Saudi Arabia for different front-of-pack labelling approaches.

## Figures and Tables

**Table 1:** Number of products included in each nutrient profile model’s analysis and overall.

Category	Saudi Arabia NPM	Nutri-Score	Chile HFSS	All 3 NPMs
Baked Goods	237	236	241	236
Bottled Water	10	14	14	10
Breakfast Cereals	112	112	112	112
Carbonates	16	31	31	16
Concentrates	-	-	38	-
Confectionery	540	540	567	540
Dairy	359	358	362	358
Edible Oils	-	64	73	-
Energy Drinks	10	10	10	10
Ice Cream	32	32	32	32
Juice	264	264	278	264
Other Hot Drinks	109	108	201	108
Processed Fruit and Vegetables	133	133	178	133
Processed Meat, Seafood and Alternatives	199	199	209	199
Ready-To-Drink Coffee	26	26	26	26
Ready-To-Drink Tea	24	24	24	24
Ready Meals	79	79	79	79
Rice, Pasta and Noodles	127	159	161	126
Sauces, Dips and Condiments	197	197	338	197
Savory Snacks	517	517	522	517
Soup	17	17	17	17
Sports Drinks	2	2	2	2
Sweet Biscuits, Snack Bars and Fruit Snacks	460	460	465	460
Sweet Spreads	82	82	112	82
Total	3552	3664	4092	3548

NPM = nutrient profile model; HFSS = high fat, salt, sugar.

**Table 2:** Proportion of products eligible under each nutrient profile model.

Category	Total *n*	Saudi Arabia NPM	Nutri-Score	Chile HFSS	% Agreement	Fleiss’ Kappa
Baked Goods	236	13%	14%	10%	92%	0.76
Bottled Water	10	90%	90%	100%	90%	0.46
Breakfast Cereals	112	26%	37%	10%	72%	0.50
Carbonates	16	69%	94%	88%	75%	0.40
Confectionery	540	9%	10%	9%	99%	0.94
Dairy	358	31%	25%	22%	73%	0.54
Energy Drinks	10	20%	20%	20%	100%	1.00
Ice Cream	32	6%	47%	28%	56%	0.26
Juice	264	40%	24%	13%	63%	0.35
Other Hot Drinks	108	8%	12%	6%	91%	0.62
Processed Fruit and Vegetables	133	92%	98%	91%	87%	0.25
Processed Meat, Seafood and Alternatives	199	65%	67%	63%	81%	0.72
RTD Coffee	26	15%	38%	8%	69%	0.37
RTD Tea	24	17%	33%	17%	83%	0.68
Ready Meals	79	47%	54%	56%	76%	0.68
Rice, Pasta and Noodles	126	80%	79%	71%	89%	0.79
Sauces, Dips and Condiments	197	18%	22%	14%	89%	0.76
Savory Snacks	517	6%	12%	10%	85%	0.41
Soup	17	94%	94%	94%	100%	1.00
Sports Drinks	2	50%	100%	50%	50%	0.25
Sweet Biscuits, Snack Bars and Fruit Snacks	460	10%	12%	11%	97%	0.89
Sweet Spreads	82	0%	21%	0%	79%	−0.07
Total	3548	25%	27%	22%	86%	0.74

NPM = nutrient profile model; HFSS = high fat, salt, sugar. Note: Products in this analysis were limited to products that were able to be analyzed under all three NPMs.

## Data Availability

Data were provided under license from Mintel and are not able to be shared.

## Supplementary Materials

The following supporting information can be downloaded at https://www.mdpi.com/article/10.3390/nu17111895/s1: Figure S1: Proportion of Saudi Arabian packaged food and beverage products eligible under the Saudi Arabian nutrient profile model; Figure S2: Proportion of Saudi Arabian packaged food and beverage products meeting each rating under the Nutri-Score nutrient profile model; Figure S3: Proportion of Saudi Arabian packaged food and beverage products meeting nutrient and ingredient criteria under the Chilean HFSS model; Table S1: Chile’s High Fat Salt Sugar nutrient criteria; Table S2: Missing data, by category; Table S3: Agreement between the Saudi Arabian nutrient profile model and Nutri-Score; Table S4: Agreement between the Saudi Arabian nutrient profile model and Chilean HFSS model; Table S5: Agreement between Chile’s HFSS NPM and Nutri-Score.
