# Supplementary material for: The Healthiness of Packaged Food and Beverage Products in the Kingdom of Saudi Arabia
Source: Nutrients. 2025 May 31;17(11):1895. doi: 10.3390/nu17111895 (PMC12158256; doi:10.3390/nu17111895)
Supplement: Supplementary file 1 [file nutrients-17-01895-s001.zip › nutrients-3654695-supplementary.pdf]

Supplementary Material

Supplementary Table S1: Chile's nutrient criteria

Solid food	Phase 1	Phase 2	Phase 3
Energy (kcal/100g)	350	300	275
Sodium (mg/100g)	800	500	400
Total sugars (g/100g)	22.5	15.0	10.0
Saturated fat (g/100g)	6.0	5.0	4.0
**Liquids**	**Phase 1**	**Phase 2**	**Phase 3**
Energy (kcal/100mL)	100	80	70
Sodium (mg/100mL)	100	100	100
Total sugars (g/100mL)	6.0	5.0	5.0
Saturated fat (g/100mL)	3.0	3.0	3.0

Supplementary Table S2: Missing data, by category

Category	Total n	% missing Saturated fat	% missing total sugar	% missing sodium
**Baby Foods**	69	57%	52%	9%
**Baked Goods**	317	16%	20%	18%
**Bottled Water**	100	84%	83%	7%
**Breakfast Cereals**	131	8%	8%	11%
**Carbonates**	240	15%	23%	25%
**Concentrates**	53	21%	17%	25%
**Confectionery**	703	14%	14%	14%
**Dairy**	741	39%	44%	39%
**Edible Oils**	193	28%	26%	30%
**Energy Drinks**	14	0%	0%	29%
**Ice Cream**	78	56%	55%	59%
**Juice**	458	21%	26%	32%
**Other Hot Drinks**	380	42%	44%	41%
**Processed Fruit and Vegetables**	292	33%	29%	24%
**Processed Meat, Seafood and Alternatives**	510	49%	50%	39%
**RTD Coffee**	33	9%	9%	21%
**RTD Tea**	26	0%	8%	8%
**Ready Meals**	130	22%	34%	15%
**Rice, Pasta and Noodles**	264	32%	31%	28%
**Sauces, Dips and Condiments**	750	39%	39%	37%
**Savoury Snacks**	663	11%	15%	14%
**Soup**	23	0%	4%	22%
**Sports Drinks**	5	0%	60%	0%
**Sweet Biscuits, Snack Bars and Fruit Snacks**	570	14%	14%	13%
**Sweet Spreads**	197	24%	40%	29%
**Total**	**6,940**	**28%**	**30%**	**26%**

Supplementary Table S3: Agreement between the Saudi Arabia Nutrient Profile Model and Nutri-Score

Category	Total n	Saudi Arabia NPM	Nutri-Score	% agreement	Fleiss' kappa
**Baked Goods**	236	13%	14%	97%	0.85
**Bottled Water**	10	90%	90%	100%	1.00
**Breakfast Cereals**	112	26%	37%	88%	0.71
**Carbonates**	16	69%	94%	75%	0.26
**Confectionery**	540	9%	10%	99%	0.93
**Dairy**	358	31%	25%	78%	0.45
**Energy Drinks**	10	20%	20%	100%	1.00
**Ice Cream**	32	6%	47%	59%	0.14
**Juice**	264	40%	24%	79%	0.53
**Other Hot Drinks**	108	8%	12%	93%	0.60
**Processed Fruit and Vegetables**	133	92%	98%	94%	0.32
**Processed Meat, Seafood and Alternatives**	199	65%	67%	94%	0.87
**RTD Coffee**	26	15%	38%	77%	0.45
**RTD Tea**	24	17%	33%	83%	0.57
**Ready Meals**	79	47%	54%	87%	0.75
**Rice, Pasta and Noodles**	126	80%	79%	98%	0.93
**Sauces, Dips and Condiments**	197	18%	22%	95%	0.84
**Savoury Snacks**	517	6%	12%	94%	0.62
**Soup**	17	94%	94%	100%	1.00
**Sports Drinks**	2	50%	100%	50%	0.00
**Sweet Biscuits, Snack Bars and Fruit Snacks**	460	10%	12%	98%	0.89
**Sweet Spreads**	82	0%	21%	79%	0.00
**Total**	**3548**	**25%**	**27%**	**91%**	**0.78**

Products in this analysis were limited to products that were able to be analyzed under both NPMs

Supplementary Table S4: Agreement between the Saudi Arabia Nutrient Profile Model and Chile HFSS model

Category	Total n	Saudi Arabia NPM	Chile HFSS	% agreement	Fleiss' kappa
**Baked Goods**	237	13%	10%	94%	0.71
**Bottled Water**	10	90%	100%	90%	0.00
**Breakfast Cereals**	112	26%	10%	84%	0.48
**Carbonates**	16	69%	88%	81%	0.48
**Confectionery**	540	9%	9%	99%	0.94
**Dairy**	359	31%	21%	79%	0.46
**Energy Drinks**	10	20%	20%	100%	1.00
**Ice Cream**	32	6%	28%	72%	0.09
**Juice**	264	40%	13%	70%	0.28
**Other Hot Drinks**	109	8%	6%	95%	0.60
**Processed Fruit and Vegetables**	133	92%	91%	88%	0.21
**Processed Meat, Seafood and Alternatives**	199	65%	63%	83%	0.64
**RTD Coffee**	26	15%	8%	92%	0.63
**RTD Tea**	24	17%	17%	100%	1.00
**Ready Meals**	79	47%	56%	78%	0.57
**Rice, Pasta and Noodles**	127	80%	71%	88%	0.69
**Sauces, Dips and Condiments**	197	18%	14%	93%	0.76
**Savoury Snacks**	517	6%	10%	88%	0.21
**Soup**	17	94%	94%	100%	1.00
**Sports Drinks**	2	50%	50%	100%	1.00
**Sweet Biscuits, Snack Bars and Fruit Snacks**	460	10%	11%	99%	0.93
**Sweet Spreads**	82	0%	0%	100%	1.00
**Total**	**3552**	**25%**	**22%**	**89%**	**0.72**

Products in this analysis were limited to products that were able to be analyzed under both NPMs

Supplementary Table S5: Agreement between Chile's HFSS NPM and Nutri-Score

Category	Total n	Chile HFSS	Nutri-Score	% agreement	Fleiss' kappa
**Baked Goods**	236	10%	14%	94%	0.72
**Bottled Water**	14	100%	93%	93%	0.00
**Breakfast Cereals**	112	10%	37%	73%	0.32
**Carbonates**	31	90%	94%	97%	0.78
**Confectionery**	540	9%	10%	99%	0.95
**Dairy**	358	22%	25%	90%	0.72
**Edible Oils**	64	19%	94%	22%	-0.01
**Energy Drinks**	10	20%	20%	100%	1.00
**Ice Cream**	32	28%	47%	81%	0.61
**Juice**	264	13%	24%	78%	0.28
**Other Hot Drinks**	108	6%	12%	94%	0.67
**Processed Fruit and Vegetables**	133	91%	98%	92%	0.27
**Processed Meat, Seafood and Alternatives**	199	63%	67%	84%	0.66
**RTD Coffee**	26	8%	38%	69%	0.24
**RTD Tea**	24	17%	33%	83%	0.57
**Ready Meals**	79	56%	54%	86%	0.72
**Rice, Pasta and Noodles**	159	77%	84%	93%	0.78
**Sauces, Dips and Condiments**	197	14%	22%	90%	0.68
**Savoury Snacks**	517	10%	12%	88%	0.38
**Soup**	17	94%	94%	100%	1.00
**Sports Drinks**	2	50%	100%	50%	0.00
**Sweet Biscuits, Snack Bars and Fruit Snacks**	460	11%	12%	97%	0.85
**Sweet Spreads**	82	0%	21%	79%	0.00
**Total**	**3664**	**23%**	**29%**	**89%**	**0.72**

Products in this analysis were limited to products that were able to be analyzed under both NPMs

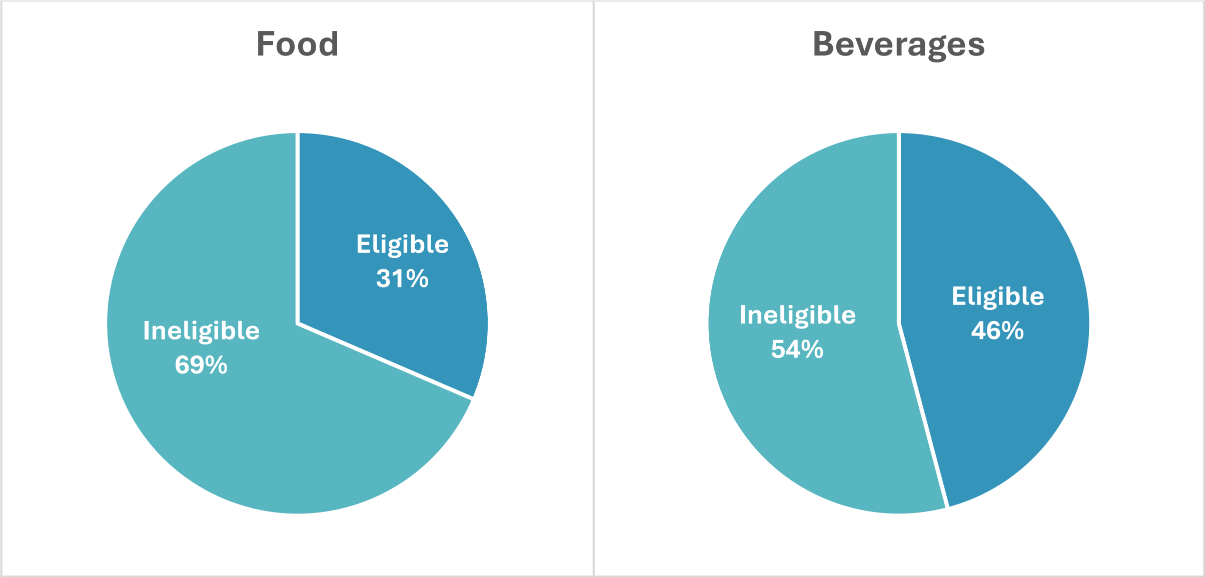

Supplementary Figure S1: Proportion of Saudi Arabian packaged food and beverage products eligible under the Saudi Arabian Nutrient Profile Model

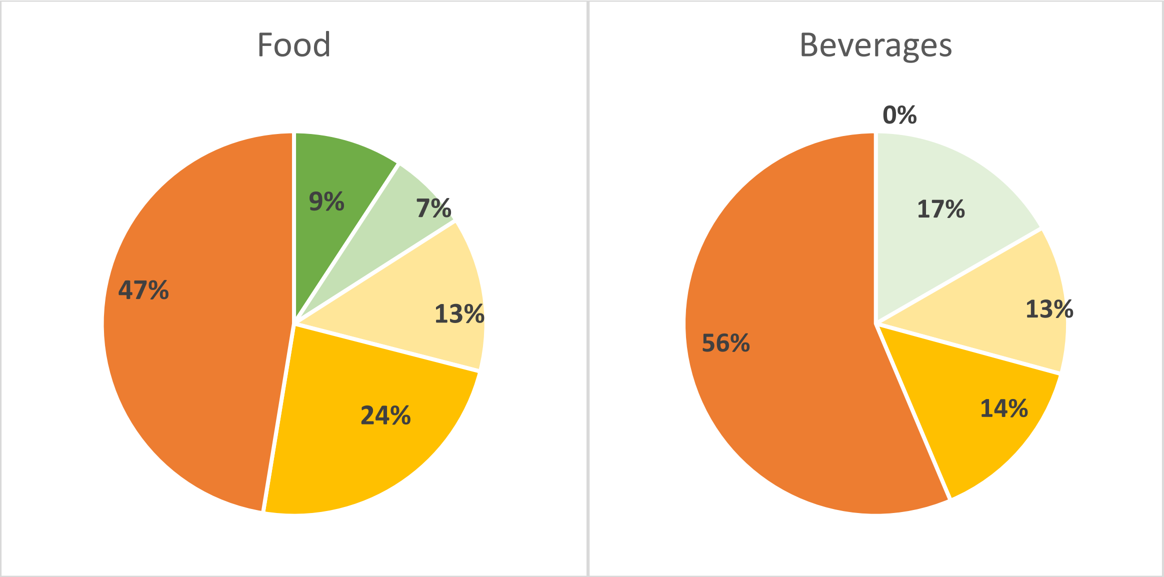

Supplementary Figure S2: Proportion of Saudi Arabian packaged food and beverage products meeting each rating under the Nutri-Score Nutrient Profile Model

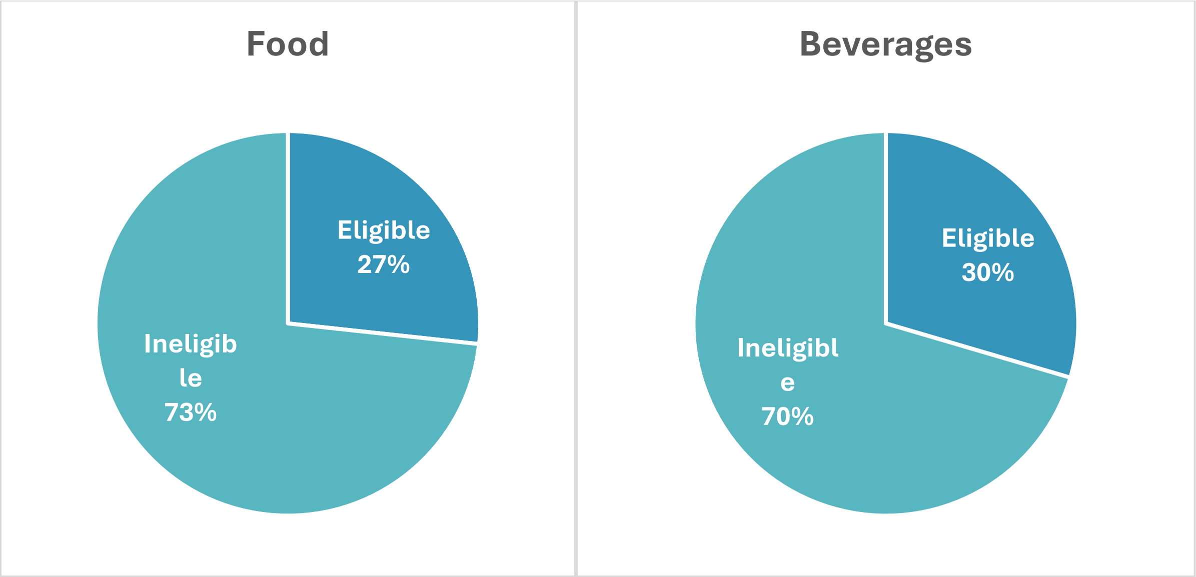

Supplementary Figure S3: Proportion of Saudi Arabian packaged food and beverage products meeting nutrient and ingredient criteria under the Chilean HFSS model
